# Receptor specificity and erythrocyte binding preferences of avian influenza viruses isolated from India

**DOI:** 10.1186/1743-422X-9-251

**Published:** 2012-10-30

**Authors:** Shailesh D Pawar, Saurabh S Parkhi, Santosh S Koratkar, Akhilesh C Mishra

**Affiliations:** 1 National Institute of Virology (NIV)-Microbial Containment Complex (MCC), 130/1, Sus Road, Pashan, Pune, 411021, India

**Keywords:** Receptor specificity, Erythrocyte binding, Avian influenza, India

## Abstract

**Introduction:**

Hemagglutination (HA) and hemagglutination inhibition (HI) assays are conventionally used for detection and identification of influenza viruses. HI assay is also used for detection of antibodies against influenza viruses. Primarily turkey or chicken erythrocytes [red blood cells (RBCs)] are used in these assays, as they are large, nucleated, and sediment fast, which makes it easy to determine the titer. Human influenza viruses agglutinate RBCs from chicken, human, and guinea pig, but not from horse. Human influenza viruses bind preferentially to sialic acid (SA) linked to galactose (Gal) by α 2, 6 linkage (SA α 2, 6-Gal), whereas avian influenza (AI) viruses bind preferentially to SA α 2, 3-Gal linkages. With this background, the present study was undertaken to study erythrocyte binding preferences and receptor specificities of AI viruses isolated from India.

**Materials and methods:**

A total of nine AI virus isolates (four subtypes) from India and three reference AI strains (three subtypes) were tested in HA and HI assays against mammalian and avian erythrocytes. The erythrocytes from turkey, chicken, goose, guinea pig and horse were used in the study. The receptor specificity determination assays were performed using goose and turkey RBCs. The amino acids present at 190 helix, 130 and 220 loops of the receptor-binding domain of the hemagglutinin protein were analyzed to correlate amino acid changes with the receptor specificity.

**Results:**

All tested highly pathogenic avian influenza (HPAI) H5N1 viruses reacted with all five types of RBCs in the HA assay; AI H9N2 and H5N2 viruses did not react with horse RBCs. For H5N1 viruses guinea pig and goose RBCs were best for both HA and HI assays. For H9N2 viruses, guinea pig, fowl and turkey RBCs were suitable. For other tested AI subtypes, avian and guinea pig RBCs were better. Eight isolates of H5N1, one H4N6 and one H7N1 virus showed preference to avian sialic acid receptors. Importantly, two isolates of HPAI H5N1, H9N2 and H11N1 viruses showed receptor specificity preference to both avian and mammalian sialic acid (α-2, 3 and α-2, 6) receptors.

**Conclusions:**

Use of different types of RBCs resulted in titer variations in HA and HI assays. This showed that RBCs giving optimum HA and HI titers would increase sensitivity of detection and would be more appropriate for identification and antigenic analysis of AI viruses. Analysis of 16 amino acids in the receptor-binding domain of the hemagglutinin of HPAI H5N1 viruses revealed that the only variation observed was in S221P amino acid position. Two H5N1 viruses showed S221P amino acid change, out of which only one H5N1 virus showed preference to α 2, 6 sialic acid receptor. One H5N1 virus isolate with amino acid S at 221 position, showed preference to α 2,3 as well as α 2,6 sialic acid receptors. This indicated that factor(s) other than S221P mutation in the hemagglutinin are probably involved in determining receptor specificity of H5N1 viruses. This is the first report of receptor specificity and erythrocyte binding preferences of AI viruses from India.

## Introduction

Highly Pathogenic Avian Influenza (HPAI) H5N1 virus emerged in Hong Kong in 1997 and since then has been threatening the poultry industry and human health worldwide [1]. In February 2006, HPAI H5N1 virus was first reported in India and since then more than seventy outbreaks of HPAI H5N1 viruses have been reported in poultry during 2006–2011 [2]. Hemagglutination (HA) and hemagglutination inhibition (HI) assays have been conventionally used for detection and identification of either tissue culture or egg-grown influenza viruses. HI assay is also used for detection of antibodies against influenza viruses. Primarily turkey or chicken erythrocytes [red blood cells (RBCs)] are used in these assays, as they are large, nucleated, and sediment fast which makes it easy to determine the titer. Human influenza viruses agglutinate RBCs from chicken, human, and guinea pig, but not from horse.

Influenza viruses bind to the sialic acid (SA) linked to galactose (Gal) on host cells through the receptor-binding site of the haemagglutinin protein [3]. Human influenza viruses bind preferentially to SA linked to Gal by α 2, 6 linkage (SA α 2, 6-Gal), whereas avian influenza (AI) viruses bind preferentially to SA α 2, 3-Gal linkages [4]. Turkey and chicken RBCs express a mixture of mainly SA α 2, 3-Gal and SA α 2, 6-Gal linkages, whereas horse RBCs contain almost exclusively SA α 2, 3-Gal linkages [5-8]. Receptor specificity of influenza A viruses correlates with their ability to agglutinate erythrocytes from different avian and mammalian species. Therefore, erythrocytes from different hosts can be used to rapidly define the receptor specificity of influenza A viruses [9]. The efficiency of haemagglutinin binding is dependent on the type of SA and linkage that connects the SA residue with the oligosaccharide of the receptor molecule [10].

Use of horse RBCs has been recommended for HA and HI assays of AI H5N1 viruses [11]. Studies have shown that goose RBCs confer a greater advantage over erythrocytes of other species in both HA and HI assays for AI viruses. Wiriyarat et. al. have shown that guinea pig and goose RBCs were best for HA assays of AI viruses [12]. The presence of SA α 2,6-Gal but not SA α 2,3-Gal on the surface of epithelial cells in human trachea underscores the importance of role of receptor specificity in the host range restriction of influenza viruses [9]. With this background the present study was undertaken to determine receptor specificity and erythrocyte binding preferences of HPAI H5N1, H9N2, H11N1 and H4N6 viruses isolated from India.

## Materials and methods

### Viruses used

A total of nine AI viruses isolated from India (four subtypes) and three reference strains (three subtypes) from OIE, Italy were used in HA and HI assays. A total of 13 AI isolates (four subtypes) from India and one reference H7N1 strain was used in receptor specificity determination assays (Table 1). The Indian AI virus isolates belonged to five states of India namely Maharashtra, Manipur, West Bengal, Assam and Tripura which were isolated and characterized at the National Institute of Virology (NIV) Pune, India. AI H9N2, H4N6, H11N1 were low pathogenic avian influenza (LPAI) viruses. AI H5N2 (A/Turkey/Italy/80) and H7N1 (A/Chicken/Italy/1067/V99) were inactivated reference viruses obtained from the OIE/FAO National Reference Laboratory for AI and Newcastle disease, Legnaro, Italy.

**Table 1:** Receptor specificity determination assay results of five subtypes of avian influenza viruses with and without α 2, 3-sialidase-enzyme treatment of RBCs

**No.**	** Virus strain**	**HA titer with enzyme treated 1% goose RBCs**	**HA titer with untreated 1% goose RBCs**	**HA titer with enzyme treated 0.5% turkey RBCs**	**HA titer with untreated 0.5% turkey RBCs**	**Receptor specificity Avian / mammalian**	**Amino acid present at position 221**
1	A/chicken/India/NIV33487/06-RG-2008 (H5N1)	No titer	512	No titer	512	SA α 2,3 Gal –Avian	S
2	A/chicken/India/WB-NIV2665/2008 (H5N1)	16	16	16	16	SA α 2,3 Gal and α 2, 6 Gal- Avian and Mammalian	P
3	A/chicken/India/WB-NIV2670/2008 (H5N1)	No titer	32	No titer	128	SA α 2,3 Gal–Avian	P
4	A/chicken/Manipur/NIV9743/2007 (H5N1)	No titer	8	No titer	24	SA α 2,3 Gal–Avian	S
5	A/chicken/India/WB-NIV2811/2008 (H5N1)	No titer	24	No titer	256	SA α 2,3 Gal–Avian	S
6	A/chicken/India/AS-NIV15983/2008 (H5N1)	8	192	16	512	SA α 2,3 Gal and α 2, 6 Gal-Avian and Mammalian	S
7	A/duck/India/TR-NIV4396/2008 (H5N1)	No titer	32	No titer	24	SA α 2,3 Gal–Avian	S
8	A/chicken/India-WB-NIV82853/2008 (H5N1)	No titer	16	No titer	768	SA α 2,3 Gal–Avian	S
9	A/chicken/India/AS-NIV15618/2008 (H5N1)	No titer	48	No titer	32	SA α 2,3 Gal–Avian	S
10	A/chicken/India/AS-NIV16717/2008 (H5N1)	No titer	16	No titer	16	SA α 2,3 Gal–Avian	S
11	A/chicken/Pune/099321/2009 (H9N2)	512	1024	512	1024	SA α 2,3 Gal and α 2, 6 Gal- Avian and Mammalian	P
12	A/aquatic bird/India/NIV-17095/2007 (H11N1)	1024	1024	512	1024	SA α 2,3 Gal and α 2, 6 Gal- Avian and Mammalian	P
13	A/Duck/Nabagram-WB/101018/2009 (H4N6)	No titer	512	No titer	128	SA α 2,3 Gal–Avian	P
14	A/chicken/Italy/1067/1999 (H7N1)	No titer	512	No titer	512	SA α 2,3 Gal–Avian	P

### RBCs used

Blood from turkey, chicken, goose and guinea pig was collected aseptically in Alsever’s solution at 1:3 ratio and kept at 4°C. Blood from horse was collected in acid citrate dextrose (ACD) solution at 1:7 ratio and kept at 4°C until use. The Institutional Animal Ethical Committee (IAEC) approved the animal experiments. The RBCs were washed thrice and working suspensions of chicken (0.5%), turkey (0.5%) and goose (1%), horse (1%) and guinea pig (0.75%) were prepared. All RBCs were prepared in phosphate buffered saline (PBS) pH 7.2 [12].

### Pre-treatment of reference sera for HI assay

Serum samples were incubated with receptor-destroying enzyme (RDE) [Denka Seiken Co., Ltd] at 1:3 ratio at 37°C in a water bath overnight. RDE was subsequently inactivated by heating at 56°C in a water bath for 30 minutes to remove the non-specific inhibitors and six parts of PBS was added to make final dilution 1:10 [13]. All HA and HI assays were performed twice and samples were tested in duplicates in each assay.

### HA and HI assays

HA and HI assays were performed as described previously in the WHO manual on animal influenza diagnosis and surveillance with few modifications [13].

### HA assay

Briefly, 50 μl of PBS (pH = 7.2) was taken in all wells of the micro titer plates. In the first row 50 μl of the test sample was taken, and serially diluted by transferring 50 μl from the first well to the successive well and so on. 50 μl of the RBC suspension was added to each well on the plate. Cell and virus controls were kept in the same plate. Plate was incubated at room temperature (R.T.). Titers were recorded after 30 minutes for avian RBCs, 45 minutes for guinea pig and one hour for horse RBCs. Hemagglutination units were expressed as the reciprocal of the maximum dilution of virus that resulted in complete agglutination [13].

### HI assay

A total of seven immune sera against AI viruses were used. Reference immune sera obtained from the OIE/FAO National Reference Laboratory for AI and Newcastle disease, Legnaro, Italy; WHO and the standard fowl sera against the AI viruses prepared at NIV were used. Virus HA titration was carried out and 8 HA units (HAU) virus was prepared (4 HAU/25 μl). 25 μl of PBS was taken in all wells of micro-titer plate. 25 μl of RDE treated serum (1:10 dilution) was added to the first well and was serially diluted up to the last well. Standard antigen of 4 HAU/25 μl was added in all wells except cell controls. Plates were incubated at R.T. for 30 minutes for antigen and antibody reaction. 50 μl of the RBC suspension was added in all wells including the cell control, serum and virus control wells. Plates were incubated at R.T. for 30–45 minutes and results were recorded. Geometric mean titers (GMTs) of H5N1 and H9N2 virus subtypes were calculated to compare performance of various RBCs. GMTs were not calculated for other AI subtypes, as only one strain of each subtype was available for the study. A total of four readings of the each sample in HA and HI assays were used to calculate GMT.

### Alpha 2, 3-sialidase assay

This assay was done to determine receptor specificity of AI viruses using goose (1%) and turkey (0.5%) RBCs. Sialidase treated and un-treated goose and turkey RBCs were tested against 13 representative Indian isolates of AI viruses and one reference strain of AI H7N1 were used. The treatment of RBCs by alpha 2, 3-sialidase enzyme specifically cleaved alpha 2, 3-sialyl linkages from glycoproteins and glycolipids. The assay was performed as per Auewarakul *et al*. [14]. Briefly, 50 μl of 10% RBC suspension prepared in PBS was treated with 1.25 units of alpha 2,3-sialidase enzyme [Takara Bio Inc.] for 1 hour at 37°C. These sialidase enzyme-treated RBCs were used in HA assay.

### Analysis of amino acids (H3 numbering) at the receptor-binding domain of the hemagglutinin protein

The sequences of the hemagglutinin gene of the AI viruses included in the study were downloaded from the NCBI database (Accession nos. EF362418, CY046081, CY046083, FJ719834, CY046093, GQ917223, CY046102, GU083637, CY055175, JX310062, AJ584647). The HA gene of AI H9N2 and H4N6 were also included in the study (kindly provided by Dr. J. Mullick, NIV, Pune, India). Sequence analysis was performed using BioEdit sequence alignment editor and H3 numbering system was followed for the amino acid analysis. The receptor binding domain positions [190 helix; amino acid position 188 to 190, 130-loop; amino acid position 134–138 and 220-loop; amino acid position 221–228] were analyzed to correlate amino acid changes with receptor specificity of AI virus isolates.

## Results

For HPAI H5N1 viruses, guinea pig, goose and turkey erythrocytes were best for HA assay; guinea pig and horse RBCs were best for HI assay. For AI H9N2 viruses, guinea pig, fowl and turkey erythrocytes were best. For other tested AI subtypes, avian and guinea pig erythrocytes were better for HA and HI assays. AI H9N2 and H5N2 viruses did not react with horse RBCs (Tables 2, 3, 4, 5).

**Table 2:** Hemagglutination titers of H5N1 and H9N2 AI viruses with five different types of RBCs

**Sr. No.**	**Virus strains**	**RBCs**				
		**Turkey (0.5%)**	**Fowl (0.5%)**	**Goose (1%)**	**Guinea Pig (0.75%)**	**Horse (1%)**
		**HA titer**				
1	A/chicken/Navapur/India/7972/2006 (H5N1)	64	32	64	128	64
2	A/chicken/Manipur/NIV9743/2007 (H5N1)	64	64	64	256	64
3	A/chicken/India/AS-NIV15983/2008/11/27 (H5N1)	256	128	512	512	8
4	A/chicken/India/WB-NIV2811/2008 (H5N1)	512	256	1024	1024	512
5	A/chicken/India/NIV33487/06-RG-2008 (H5N1)	64	48	48	24	16
	**Geometric mean titer**	**128.0**	**79.7**	**159.4**	**210.4**	**48.5**
1	A/chicken/Pune/099321/2009 (H9N2)	2048	2048	512	2048	No titer
2	A/chicken/West Bengal/1057183/2010 (H9N2)	1024	768	512	768	No titer
3	A/Turkey/Wisconsin/66 (H9N2)-OIE	4	8	4	8	No titer
	**Geometric mean titer**	**203.2**	**232.6**	**101.6**	**232.6**	**-**

**Table 3:** Hemagglutination titers of four AI viruses with five different types of RBCs

**Sr. No.**	**Virus strains**			**RBCs**		
		**Turkey (0.5%)**	**Fowl (0.5%)**	**Goose (1%)**	**Guinea Pig (0.75%)**	**Horse (1%)**
				**HA titer**		
1	A/turkey/Italy/80 (H5N2)-OIE	32	32	16	64	No titer
2	A/chicken/Italy/1067/1999 (H7N1)-OIE	192	512	128	128	64
3	A/aquatic bird/India/NIV-17095/2007 (H11N1)	384	384	128	384	512
4	A/duck/Nabagram-WB/101018/2009 (H4N6)	64	64	64	64	64

**Table 4:** Hemagglutination inhibition (HI) titers of immune/reference sera against H5N1 viruses in HI assay with five different types of RBCs

**Sr. No.**	**Virus strain**	**Antisera**	**RBCs**				
			**Turkey (0.5%)**	**Fowl (0.5%)**	**Goose (1%)**	**Guinea Pig (0.75%)**	**Horse (1%)**
				**HI titer**			
1	A/chicken/Navapur/India/7972/2006 (H5N1)	H5N1-NIV	320	160	320	320	1280
		H5N1-OIE	20	20	20	20	20
		H5N1-WHO	320	80	160	320	640
2	A/chicken/Manipur/NIV9743/2007 (H5N1)	H5N1-NIV	No titer	20	No titer	160	640
		H5N1-OIE	20	No titer	No titer	20	20
		H5N1-WHO	160	160	320	640	320
3	A/chicken/India/AS-NIV15983/ 2008/11/27 (H5N1)	H5N1-NIV	1280	640	2560	1280	320
		H5N1-OIE	80	40	320	320	20
		H5N1-WHO	1280	640	2560	1280	160
4	A/chickenIndia/WB-NIV2811/2008 (H5N1)	H5N1-NIV	2560	640	2560	5120	5120
		H5N1-OIE	80	160	40	1280	640
		H5N1-WHO	1280	640	1280	5120	5120
5	A/chicken/India/NIV33487/06-RG-2008 (H5N1)	H5N1-NIV	320	320	640	480	480
		H5N1-OIE	160	240	320	240	480
	**Geometric mean titer**		**245.1**	**165.0**	**403.2**	**434.3**	**339.0**

**Note:** For calculation of geometric mean antibody titers, value for “No titer” was 5.

**Table 5:** Hemagglutination inhibition (HI) titers of immune/reference sera against four subtypes of AI viruses in HI assay with five different types of RBCs

**Sr. No.**	** Virus antigens**	** Antisera**	**RBCs**				
			**Turkey (0.5%)**	**Fowl (0.5%)**	**Goose (1%)**	**Guinea Pig (0.75%)**	**Horse (1%)**
			**HI titer**				
1	A/turkey/Italy/80 (H5N2)-OIE	H5-OIE	560	2560	2560	1280	No titer
2	A/chicken/Italy/1067/1999 (H7N1)-OIE	H7N1-OIE	640	640	640	320	320
3	A/aquatic bird/India/NIV-17095/2007 (H11N1)	H11N1-NIV	1280	1280	1280	1280	1280
4	A/chicken/Pune/099321/2009 (H9N2)	H9N2-NIV	180	1280	1280	180	No titer
5	A/chicken/West Bengal/1057183/2010 (H9N2)	H9N2-NIV	1280	1280	1280	1280	No titer

In sialidase assay, eight HPAI H5N1 viruses, H4N6 and H7N1 did not show hemagglutination, showing avian receptor (SA α 2, 3-Gal) specificity. Two isolates of H5N1, H11N1 and H9N2 viruses showed hemagglutination even after cleaving alpha 2, 3 sialic acid linked receptors from turkey and goose RBCs, showing binding preferences to both avian (SA α 2,3-Gal) and mammalian sialic acid (SA α 2,6-Gal) receptors. All H5N1 virus strains showed conserved receptor binding domains, except for two strains (WB-NIV-2665 and WB-NIV-2670) which have S221P amino acid change, out of which only one H5N1 virus showed preference to α 2, 6 sialic acid receptor. Another H5N1 virus (AS-NIV-15983) with amino acid S at 221 position in hemagglutinin protein, showed binding preference to both α 2,3 and α 2,6 sialic acid receptors. AI H9N2, H11N1, H4N6 and H7N1 viruses showed amino acid P at 221 position. AI H9N2 and H11N1 viruses showed binding preference to α 2, 3 and α 2, 6 sialic acid receptors (Table 1).

## Discussion

The present study showed that horse RBCs were not the choice of RBCs for detection and identification of AI viruses. AI H9N2 and H5N2 viruses did not react with horse RBCs both in HA and HI assays. It has been reported that horse RBCs require more viral particles for agglutination than do turkey RBCs. Similarly, using horse RBCs in HI assays require more virus than HI assay with other avian erythrocytes [11].Guinea pig RBCs were suitable for both HA and HI assays for all AI virus subtypes, but reading and interpretation of results with guinea pig RBCs was difficult. Goose and other avian RBCs were the next suitable RBCs. Advantage of using avian RBCs was that reading and interpretation of results were easier than mammalian RBCs.

With different types of RBCs, variation in HA and HI titers was observed among isolates of the same subtype. Therefore to conclude overall performance or reactivity of RBCs, calculation of GMT among the same subtype was necessary to predict suitability of RBCs. Overall guinea pig, goose and turkey RBCs were best in HA and HI assays for AI viruses. These results are in agreement with the study by Witthawat *et. al.*[12]. Suda Louisirirotchanakul *et al.*[6], have reported that goose erythrocytes confer a greater advantage over other erythrocyte species in both HA and HI assays [6]. Currently, HI-based antigenic analysis is used for influenza virus strains [15]. The present study showed that use of different RBCs results in titer differences in HI assay. Therefore, RBCs giving optimum HA and HI titers would increase sensitivity of detection and would be more appropriate for identification and antigenic analysis of AI viruses.

The direct binding assays based on different gangliosides have been used previously to study the receptor specificity of influenza A and B viruses. Hemagglutination with erythrocytes, which were enzymatically modified to contain sialyloligosaccharides of defined sequences, or hemolysis assays based on ganglioside-coated erythrocytes also have been used. These methods require specific reagents and tend to be technically difficult and time-consuming [9]. In the present study, a simpler assay described by Auewarakul et. al. was employed to determine receptor specificity by using alpha 2, 3 sialidase enzyme. The sialidase enzyme theoretically cleaves the entire alpha 2, 3 sialic acid linked receptors on the RBCs [14]. Goose and turkey RBCs were used for receptor specificity assays and results of both RBCs were in agreement. In receptor specificity assays, eight H5N1 isolates, H4N6 and H7N1 viruses clearly indicated preference to avian receptors. Two H5N1 isolates, H9N2 and H11N1 viruses showed ability to bind both α 2,3 and α 2,6 sialic acid linked receptors, highlighting their potential to cross species barrier.

The amino acid 221 position of HA protein has critical role in deciding receptor specificity of H5N1 virus and change of amino acid S221P is responsible for change in avian receptor specificity [2]. All analyzed amino acids at the receptor-binding domain of hemagglutinin protein were conserved in analyzed AI virus strains except S221P change. Two H5N1 viruses showed S221P amino acid change, out of which only one H5N1 virus showed preference to α 2, 6 sialic acid receptor. One H5N1 isolate showed amino acid S at 221 position, still showed preference to α 2, 6 along with α 2, 3 sialic acid receptor. Possibility of the presence of a minor variant within the viral quasispecies that had altered receptor-binding specificity cannot be ruled out. Amino acid P was found at 221 position of hemagglutinin in AI H9N2, H11N1, H4N6 and H7N1 viruses. AI H9N2 and H11N1 virus showed receptor specificity to α 2, 3 and α 2, 6 sialic acid receptors. Involvement of factor(s) other than S221P mutation in the hemagglutinin protein in determining receptor specificity of H5N1 viruses needs further investigation. In the current scenario of emerging influenza viruses, virological as well as molecular characterization and analysis of receptor preferences of AI viruses would be important to study animal-human interface.

## Conclusions

Use of different types of RBCs resulted in titer variations in HA and HI assays. This showed that RBCs giving optimum HA and HI titers would increase sensitivity of detection and would be more appropriate for identification and antigenic analysis of AI viruses. Analysis of 16 amino acids in the receptor-binding domain of the hemagglutinin of HPAI H5N1 viruses revealed that the only variation observed was in S221P amino acid position. Two H5N1 viruses showed S221P amino acid change, out of which only one H5N1 virus showed preference to α 2, 6 sialic acid receptor. One H5N1 virus isolate with amino acid S at 221 position, showed preference to α 2,3 as well as α 2,6 sialic acid receptors. This indicated that factor(s) other than S221P mutation in the hemagglutinin are probably involved in determining receptor specificity of H5N1 viruses.

## Competing interests

The authors declare that they have no competing interests.

## Authors’ contributions

SDP and ACM conceived and designed the experimental work. SDP, SSP and SSK performed the experimental work. SDP, SSP and SSK analyzed the data. SDP, SSP and ACM wrote the paper. All authors read and approved the final manuscript.
